# A role for β-1,6- and β-1,3-glucans in kinetochore function in *Saccharomyces cerevisiae*

**DOI:** 10.1093/genetics/iyad195

**Published:** 2023-11-10

**Authors:** Rucha Kshirsagar, Arno Munhoven, Tra My Tran Nguyen, Ann E Ehrenhofer-Murray

**Affiliations:** Institut für Biologie, Humboldt-Universität zu Berlin, Philippstr. 13, Rhoda-Erdmann-Haus, 10099 Berlin, Germany; Institut für Biologie, Humboldt-Universität zu Berlin, Philippstr. 13, Rhoda-Erdmann-Haus, 10099 Berlin, Germany; Institut für Biologie, Humboldt-Universität zu Berlin, Philippstr. 13, Rhoda-Erdmann-Haus, 10099 Berlin, Germany; Institut für Biologie, Humboldt-Universität zu Berlin, Philippstr. 13, Rhoda-Erdmann-Haus, 10099 Berlin, Germany

**Keywords:** CENP-A, Cse4, Kre6, Fks1, Kre11/Trs65, Chs1

## Abstract

Chromosome segregation is crucial for the faithful inheritance of DNA to the daughter cells after DNA replication. For this, the kinetochore, a megadalton protein complex, assembles on centromeric chromatin containing the histone H3 variant CENP-A, and provides a physical connection to the microtubules. Here, we report an unanticipated role for enzymes required for β-1,6- and β-1,3-glucan biosynthesis in regulating kinetochore function in *Saccharomyces cerevisiae*. These carbohydrates are the major constituents of the yeast cell wall. We found that the deletion of *KRE6*, which encodes a glycosylhydrolase/ transglycosidase required for β-1,6-glucan synthesis, suppressed the centromeric defect of mutations in components of the kinetochore, foremost the NDC80 components Spc24, Spc25, the MIND component Nsl1, and Okp1, a constitutive centromere-associated network protein. Similarly, the absence of Fks1, a β-1,3-glucan synthase, and Kre11/Trs65, a TRAPPII component, suppressed a mutation in *SPC25*. Genetic analysis indicates that the reduction of intracellular β-1,6- and β-1,3-glucans, rather than the cell wall glucan content, regulates kinetochore function. Furthermore, we found a physical interaction between Kre6 and CENP-A/Cse4 in yeast, suggesting a potential function for Kre6 in glycosylating CENP-A/Cse4 or another kinetochore protein. This work shows a moonlighting function for selected cell wall synthesis proteins in regulating kinetochore assembly, which may provide a mechanism to connect the nutritional status of the cell to cell-cycle progression and chromosome segregation.

## Introduction

Kinetochores are megadalton protein assemblies that physically connect the chromatin at the centromeres to the microtubules. This is necessary for the correct partitioning of sister chromatids to the daughter cells during mitosis and meiosis, and errors in kinetochore attachment lead to chromosome missegregation and aneuploidy (McAinsh and Marston 2022). The kinetochore is composed of multiple subcomplexes that assemble on the centromeric chromatin (Yatskevich *et al.* 2023). At the base of this assembly are nucleosomes that contain the centromeric histone H3 variant CENP-A, which in larger eukaryotes are interspersed with canonical nucleosomes, whereas *Saccharomyces cerevisiae* has a single nucleosome containing the CENP-A homolog Cse4 (Meluh *et al.* 1998; Furuyama and Biggins 2007). The centromeric chromatin is bound by the complexes of the inner kinetochore, most notably the constitutive centromere-associated network (CCAN) (Hori *et al.* 2008), whose component CENP-C (Mif2 in *S. cerevisiae*) binds to the centromeric nucleosome (Xiao *et al.* 2017). At its chromatin-distal side, CCAN interacts with the MIND (Mis12/Mtw1) complex (Hornung *et al.* 2014), which forms an elongated, Y-shaped rod and contains the proteins Mtw1, Dsn1, Nsl1, and Nnf1 (Dimitrova *et al.* 2016). MIND in turn interacts with the NDC80 complex (NDC80c), a hetero-tetramer consisting of the two Ndc80/Nuf2 and Spc24/Spc25 dimers (Janke *et al.* 2001; Wigge and Kilmartin 2001; Cheeseman *et al.* 2006; DeLuca *et al.* 2006; Wei *et al.* 2007). The dimers each have an elongated shaft of intertwined α-helices and two globular domains on one end (Wei *et al.* 2005; Ciferri *et al.* 2008). They interact end-to-end with each other via their shafts (Valverde *et al.* 2016) to form an elongated structure that connects on its one end to the MIND complex and on its other end to the Dam ring that encircles the microtubules (Wei *et al.* 2005; Ciferri *et al.* 2008). Besides interacting with MIND, a second recruitment route for the NDC80 complex is via the Cnn1 subcomplex of CCAN, which interacts on the centromere-proximal end with centromeric DNA and on the microtubule-proximal side with NDC80 (Schleiffer *et al.* 2012) [reviewed in (Sridhar and Fukagawa 2022)].

Proper regulation of kinetochore assembly is essential for maintaining genomic stability and preventing aneuploidy. One molecular mechanism for functional regulation is through posttranslational modification of kinetochore proteins. In earlier work, we showed that the interaction between the CCAN components Okp1^CENP-Q^/Ame1^CENP-U^ and the amino-terminus of Cse4^CENP-A^ is regulated by methylation on arginine 37 and acetylation on lysine 49 of Cse4^CENP-A^ (Anedchenko *et al.* 2019). Also, Cse4^CENP-A^ is phosphorylated at several sites (Boeckmann *et al.* 2013), and phosphorylation of serine 33 regulates the deposition of Cse4^CENP-A^ at the centromere (Hoffmann *et al.* 2018).

More recently, we identified two modifications in the core region of Cse4^CENP-A^, methylation of lysine 131 and arginine 143. These modifications lie close to the entry/exit site of the DNA from the centromeric nucleosome and affect its stability. The mutation of Cse4-R143 (*cse4-R143A*) enhances the temperature-sensitive growth and chromosome segregation defect of a mutation in *SPC25* (*spc25-1*), which encodes an NDC80 component. The mutated residue in the *spc25-1* allele, L25, is located in a bundle of 3 α-helices formed by Spc25, Spc24, and Ndc80 within the junction of NDC80c (Wigge and Kilmartin 2001; Tran Nguyen *et al.* 2023). In a genetic screen, we found that mutations in the stalk of the NDC80 complex can suppress the *cse4-R143A spc25-1* defect, showing that strengthening interactions within NDC80 can compensate for the reduced stability of the centromeric nucleosome (Tran Nguyen *et al.* 2023).

**Fig. 1.**
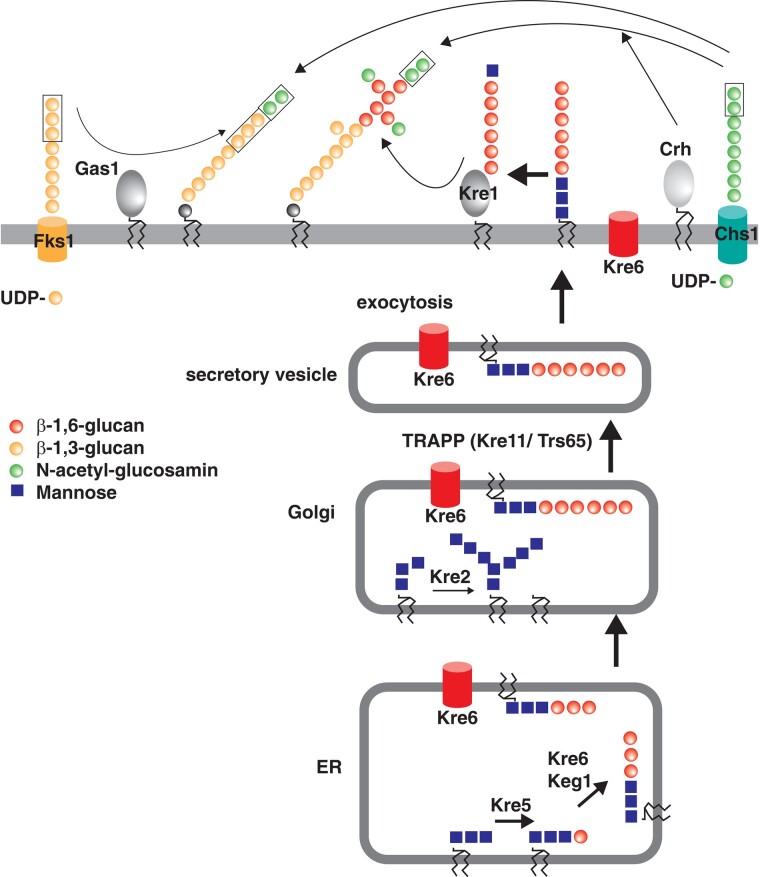
Overview of the synthesis of cell wall carbohydrate components in *S. cerevisiae*. β-1,6-glucan synthesis occurs in multiple steps from the ER via the Golgi apparatus and secretory vesicles to their attachment to cell wall proteins by Kre1. Kre11/Trs65 mediates the fusion of vesicles and the transport of β-1,6-glucan across cell compartments. Kre6 is localized in the ER, Golgi, secretory vesicles, and in the plasma membrane. β-1,3-glucan and chitin are synthesized by plasma membrane-associated β-1,3-glucan synthases, including Fks1, and chitin synthases, including Chs1, respectively, and secreted to the cell wall. The cell wall-anchored enzyme Gas1 and proteins of the Crh family rearrange polysaccharides by transferring parts of β-1,3-glucan and chitin, respectively, to existing β-1,3-, or β-1,6-glucans in the cell wall.

In the same screen, we recovered multiple isolates with mutations in *KRE6*, a gene that is required for the synthesis of β-1,6-glucan in yeast (Roemer and Bussey 1991). This led us to the unexpected discovery, reported in this work, that proteins involved in the synthesis of glucans have a role in centromere regulation. Specifically, we identified Kre6, Fks1, and Kre11/Trs65 as negative regulators of kinetochore function in *S. cerevisiae*, and Gas1 and Chs1 also played a role, though to a minor extent. These proteins have been implicated in β-glucan and chitin synthesis (Fig. 1). β-linked glucans are the major constituents of the yeast cell wall. β-1,3-glucan is the main component and is responsible for the osmotic stability of the cell. It is a branched polymer, with β-1,6 branching making up 30–80% of the cell wall mass. β-1,3-glucans are synthesized by the β-1,3-glucan synthase Fks1, which is localized to the plasma membrane and extrudes newly synthesized linear glucan through its transmembrane channel into the cell wall (Hu *et al.* 2023). The cell wall enzyme Gas1 subsequently transfers parts of β-1,3-glucans to existing β-1,3-, or β-1,6-glucans in the cell wall. Similarly, chitin (β-(1-4)-poly-N-acetyl-D-glucosamine) is synthesized by the chitin synthase Chs1, and individual units are transferred to existing chains by the Crh family of chitin transferases [reviewed in (Teparic *et al.* 2020; Ribeiro *et al.* 2022)].

Kre6 is a type II membrane protein with homology to glycosylhydrolases/transglycosidases that is localized around the nucleus, in the endoplasmic reticulum (ER), the Golgi, and at the cell periphery (Nakamata *et al.* 2007). It is required for the synthesis of β-1,6-glucan, possibly by performing cross-linking with other cell wall components, though its precise enzymatic activity is not known (Roemer and Bussey 1991; Roemer *et al.* 1993; Kurita *et al.* 2011). Kre11/Trs65 is part of the TRAPPII complex of the late Golgi, a tethering complex that mediates the interaction between transport vesicles and their acceptor compartment (Yip *et al.* 2010). *kre11Δ* cells have a β-1,6-glucan synthesis defect similar to that of *kre6Δ* (Brown *et al.* 1993), which indicates that the secretory pathway is required for β-1,6-glucan synthesis. *kre6Δ* cells are viable, but are larger than the wild-type cells and show a mild temperature sensitivity. Additionally, *kre6Δ* is synthetically lethal when *SKN1*, which encodes a Kre6 paralog, is deleted (Roemer *et al.* 1993), indicating that both Kre6 and Skn1 are required for the majority of cellular β-1,6-glucan synthesis.

**Fig. 2.**
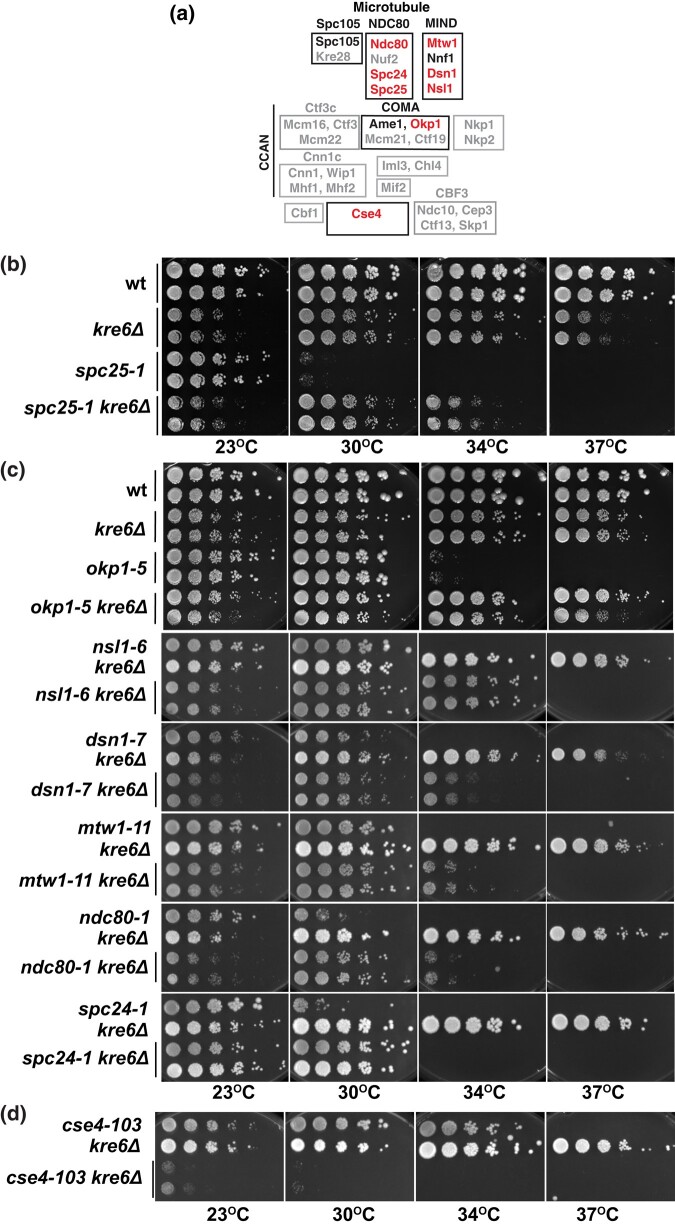
A negative role for Kre6 in kinetochore function. a) Schematic representation of the yeast kinetochore. Components indicated in red showed a synthetic genetic interaction with *kre6Δ* (Table 1). Components shown in black were tested, but showed no genetic interaction with *kre6Δ*. Gray components were not tested. The schematic does not represent protein interactions in the kinetochore. b) *kre6Δ* suppresses the temperature-sensitive growth defect of *spc25-1*. Serial dilutions of the indicated strains were spotted on YPD plates and grown for 2 days at the indicated temperatures. c) *kre6Δ* suppressed the temperature sensitivity of mutations in genes encoding components of the MIND and NDC80 complexes as well as Okp1 (a CCAN component). Serial dilutions of the respective strains were spotted on YPD and grown for 3 days at the indicated temperatures. d) *kre6Δ* enhanced the growth defect of *cse4-103*. Representation as in c).

Here, we show that β-1,6- and β-1,3-glucan synthesis participates in kinetochore regulation (Fig. 2a). Deletion of the respective biosynthesis genes suppressed the temperature sensitivity and chromosome segregation defects of a mutation in *SPC25*. *kre6Δ* furthermore showed selectivity in that it suppressed several mutant alleles of further components of the NDC80 and MIND complex. Genetic analysis of glucan biosynthesis genes indicated that the reduction of intracellular β-1,6- and β-1,3-glucan levels, but not of mannosylation or β-1,6- and β-1,3-glucans in the cell wall, was involved in kinetochore regulation. In support of a direct role, we found a physical interaction between Kre6 and Cse4^CENP-A^ in yeast cells. Altogether, this reveals an unanticipated aspect of kinetochore regulation and suggests that one or several proteins of the kinetochore are regulated by glycosylation.

## Materials and methods

### Yeast strains and plasmids

The *S. cerevisiae* strains and plasmids used in this study are listed in Supplementary Tables 1 and 2 (Supplementary File 1), respectively. Yeast was grown and manipulated according to standard procedures (Sherman 1991). Yeast was grown on full medium (YPD) and selective minimal plates. Gene deletions and epitope-tagged alleles were constructed at the endogenous loci using standard PCR-based integration and confirmed by PCR and sequence analysis (Longtine *et al.* 1998). Epitope tagging was confirmed by Western blotting. Strains with temperature-sensitive alleles combined with gene deletions were obtained by genetic crosses of deletion strains with the strains carrying the temperature-sensitive (ts) allele, and several segregants were tested for suppression of the ts allele. Strains with *cse4-103* and *kre6Δ* or *fks1Δ* were constructed by obtaining *cse4Δ kre6Δ* or *fks1Δ* strains carrying a *URA3*-marked *CSE4* plasmid by genetic crosses, and the *URA3-CSE4* plasmid was subsequently replaced by a *HIS3*-marked *cse4-103* plasmid by transformation to histidine autotrophy and subsequent counterselection for the *URA3-CSE4* plasmid on medium containing 5-fluoro-orotic acid.

Plasmid loss was measured in a wt (AEY1), *kre6Δ* (AEY7110), *spc25-1* (AEY7117), and *spc25*-1 *kre6Δ* (AEY7116) strain carrying a CEN6-*TRP1* plasmid (pAE264) as previously described (McNally and Rine 1991). For statistical analysis of biological triplicates, a 1-sided *t*-test was employed.

For FACS analysis, strains were grown in YPD at 23°C and shifted for 3 h to 30°C. 0.5 mL of exponentially growing cells were fixed with 70% ethanol and prepared for flow cytometry and staining with Sytox Green dye. 100,000 cells were analyzed using a BD Accuri C6 Flow Cytometer (Anedchenko *et al.* 2019).

### Yeast protein extracts, co-immunoprecipitation, ChIP, and Western blotting

For Western blot analysis, 8 OD of cells were harvested, washed once with TBS, and resuspended in 100 μl lysis puffer (1× PBS containing 0.1% NP-40, 1 mM EDTA, and protease inhibitor). Cells were lysed by bead-beating (using a FastPrep 5G Homogenizer MP-biomedical) for 45 s at the homogenizing intensity. Loading buffer was added to each sample, and samples were heated for 5 min to 95°C. Protein amounts equivalent to 1 OD of cells were analyzed by Western blot. Antibodies used for Western blotting were α-HA (Covance MMS-101P), α-c-Myc antibody (MA1-980), and α-β-1,3-glucan [monoclonal, Biosupplies Australia, (Meikle *et al.* 1991)].

For co-immunoprecipitation, yeast strains were grown at 30°C. 200 OD yeast cells were harvested and lysed by bead-beating in 1 mL of cold immunoprecipitates (IP) lysis buffer (50 mM HEPES, 200 mM sodium acetate, 0.25% Nonidet P-40, 1 mM EDTA, 5 mM magnesium acetate, 5% glycerol, 3 mM DTT, 1 mM PMSF, and protease inhibitors). The whole-cell lysate was cleared by centrifugation, and samples were normalized for their protein concentration before being used for the IP. An aliquot of 100 μl was taken as input control. 600 μl of each sample was incubated with 5 μl of α-myc overnight followed by 2 h incubation with 50 µl of Protein G dynabeads at 4°C. For immunoprecipitation of HA-tagged Cse4 using α-HA agarose, the resin was prewashed 5 times with lysis buffer prior to overnight incubation with lysate. 70 μl of α-HA agarose (Sigma, A2095) was added to 600 μl samples. Protein-antibody-bead/agarose conjugates were washed 3 times with lysis buffer and suspended in 50 μl of sample loading buffer (final concentration 62.5 mM Tris pH 6.8, 2% SDS, 10% glycerol, 5% 2-mercaptoethanol, and 0.001% bromophenol blue). α-Myc antibody was obtained from Thermo Scientific (MA1-980) and used at a 1:500 dilution. HA-antibody (Covance) was used at 1:250. The immunoblots were imaged on a Bio-Rad imaging system.

Chromatin immunoprecipitation was performed as described (Samel *et al.* 2012).

## Results

### Kre6 is a negative regulator of kinetochore function in *S. cerevisiae*

In earlier work, we isolated suppressors of the temperature-sensitive growth defect of yeast cells carrying the mutations *spc25-1* and *cse4-R143A* with the goal of studying the role of Cse4-R143 methylation in centromere function (Tran Nguyen *et al.* 2023). Among the 50 suppressor mutants subjected to sequence analysis to determine the causative mutation, 5 isolates carried mutations in *KRE6*, which codes for a putative glycosylhydrolase/ transglycosylase that is required for β-1,6-glucan biosynthesis in yeast (Roemer and Bussey 1991). The specific mutations were Kre6-D382G, -W425C, -S469A, -D499V, and -S714 to a stop codon. The isolation of putative suppressor mutations in *KRE6* was surprising, because a role for Kre6 as a glycosylhydrolase at the kinetochore was not expected. However, the isolation of several independent mutations prompted us to pursue the investigation of *KRE6* as a potential regulator of kinetochore function.

Given that we had isolated several alleles of *KRE6*, we hypothesized that the lack of Kre6 function caused the suppression. We therefore tested the effect of the deletion of *KRE6* (*kre6Δ*) on the growth of *spc25-1*  *cse4-R143A* and of *spc25-1* alone. As had been reported earlier (Roemer *et al.* 1993), *kre6Δ* alone caused a mild temperature sensitivity (Supplementary Fig. 1a). Importantly, *kre6Δ* suppressed the temperature-sensitive growth defect of *spc25-1*, since *spc25-1*  *kre6Δ* cells were able to grow up to a temperature of 34°C, whereas *spc25-1* cells were unable to grow at 30°C (Fig. 2b). *kre6Δ* also suppressed the temperature sensitivity of *spc25-1*  *cse4-R143A* (Supplementary Fig. 1b). Since, this indicates that the effect of *kre6Δ* is independent of Cse4-R143 modification, we did not further consider *cse4-R143A* in subsequent experiments.

Since a role for Kre6 kinetochore function was unexpected, we wondered whether the effect of *kre6Δ* on *spc25-1* was due to an indirect effect on cell wall physiology, rather than a specific effect at the kinetochore. If so, one would expect *kre6Δ* to suppress any temperature-sensitive mutation, regardless of its function at the kinetochore. To test this, *kre6Δ* was investigated for the suppression of other temperature-sensitive mutations in genes encoding kinetochore components (Fig. 2a and c). This revealed an interesting selectivity of genetic interactions. Specifically, *kre6Δ* strongly suppressed the growth defect of a mutation in *OKP1* (Ortiz *et al.* 1999). Okp1, the homolog of CENP-Q, is a component of the CCAN complex of the inner kinetochore and interacts with the N-terminus of Cse4 (Anedchenko *et al.* 2019; Fischbock-Halwachs *et al.* 2019). Furthermore, *kre6Δ* partially suppressed the defect of *nsl1-6*, and the respective protein Nsl1 is part of the MIND complex that links inner and outer kinetochore complexes. *kre6Δ* showed weaker suppression of defects caused by mutations in *DSN1* and *MTW1*, which also encode MIND components (Euskirchen 2002). Also, *kre6Δ* partially suppressed *NDC80* and *SPC24* temperature-sensitive growth defects (Fig. 2c). The respective proteins, together with Spc25, form the NDC80 complex (Janke *et al.* 2001; Wigge and Kilmartin 2001) (Fig. 2a).

In contrast, mutations in *AME1* [CCAN component (Pot *et al.* 2005)], *NNF1* [MIND component (Euskirchen 2002)], and *SPC105* [kinetochore-null complex component (Wigge *et al.* 1998)] were not affected by the additional deletion of *KRE6* (Supplementary Fig. 1c, Table 1). Furthermore, we tested the effect of *kre6Δ* on a temperature-sensitive allele of *CSE4*, *cse4-103* (Glowczewski *et al.* 2000). Surprisingly, *kre6Δ* caused a strong enhancement (rather than suppression) of the temperature sensitivity (Fig. 2d), thus displaying the opposite effect on this allele as on other kinetochore mutations.

**Table 1.** Suppression of mutations in genes encoding kinetochore components by *kre6Δ^a^*.

Kinetochore component/complex	Allele	Suppression by *kre6Δ*
Ndc80	*ndc80-1*	+
	*spc25-1*	++
	*spc24-1*	+
Mtw1	*mtw1-11*	+
	*dsn1-7*	+
	*nsl1-6*	++
	*nnf1-77*	−
Spc105	*spc105-4*	−
COMA	*okp1-5*	+++
	*ame1-4*	−
Centromeric nucleosome	*cse4-103*	Enhancement of growth defect

+++, strong suppression, ++ moderate suppression, +, mild suppression, −, no suppression.

^*a*^Suppression of temperature-sensitive growth defect caused by *kre6Δ* in combination with the indicated allele of the gene encoding the respective kinetochore component. COMA, Ctf19/ Okp1/ Mcm21/ Ame1

Altogether, these genetic interactions showed that *kre6Δ* selectively suppressed some, but not other growth defects of kinetochore mutants, arguing for a specific effect of Kre6 as a negative regulator of kinetochore function. Also, with the exception of mutation in *OKP1* (*okp1-5*), there was a trend in the suppression pattern in that several mutations of outer kinetochore components were strongly suppressed, whereas mutations of inner kinetochore components were either unaffected, and the growth defect of the *CSE4* allele was enhanced.

### *Kre6Δ* suppresses the cell cycle and minichromosome maintenance defects of *spc25-1*

**Fig. 3.**
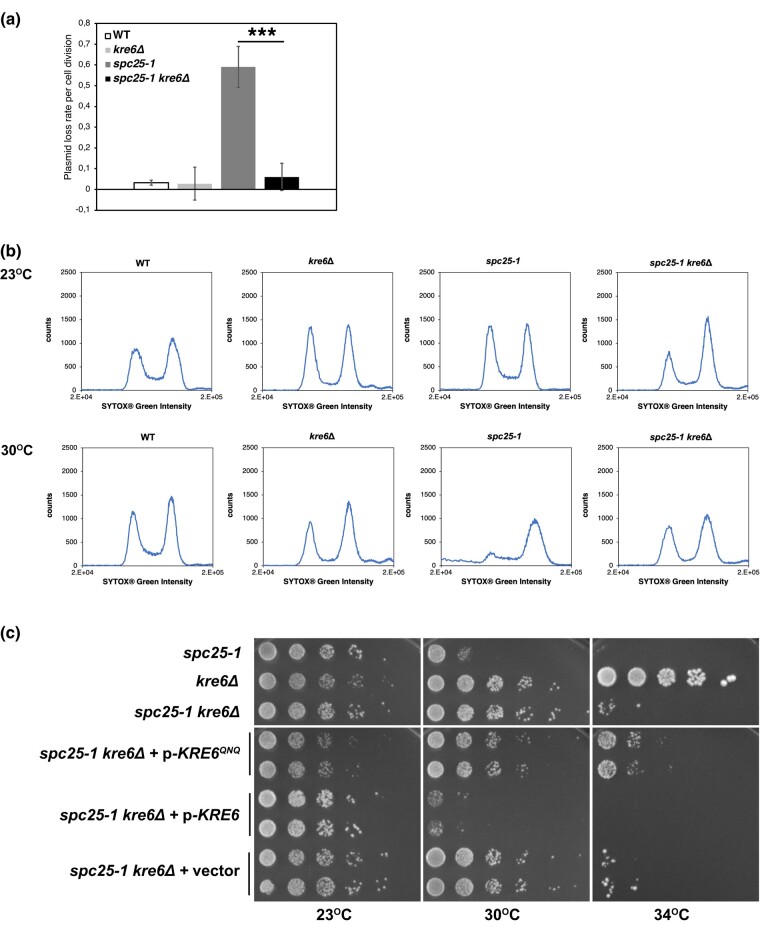
*Kre6Δ* suppressed centromeric defects of *spc25-1*. a) *kre6Δ* suppressed the plasmid maintenance defect of *spc25-1*. Error bars give a standard deviation of 6 independent experiments. ***Significant difference, *P* < 0.0001. b) *kre6Δ* suppressed the arrest of *spc25-1* cells at the G2/M phase of the cell cycle. Cells were grown to early logarithmic phase at 23°C (top row) and shifted to 30°C for 3 h (bottom row). DNA content as measured by FACS analysis is shown. c) Suppression of *spc25-1* depended on the enzymatic activity of Kre6. A *spc25-1*  *kre6Δ* strain was transformed with plasmids carrying wild-type *KRE6*, a vector control, or *KRE6*^*QNQ*^, which contains mutations in the presumed catalytic residues of Kre6. Serial dilutions were spotted on selective medium and grown for 3 days at the indicated temperatures.

The suppression of the *spc25-1* temperature sensitivity by *kre6Δ* suggested that it suppressed the chromosome segregation defect of *spc25-1*. To test this, we measured the stability of minichromosomes (plasmids) in cells that were *spc25-1* or *spc25-1*  *kre6Δ* and, as a control, in wild-type (wt) and *kre6Δ* cells. Importantly, while *spc25-1* cells showed a high rate of plasmid loss compared to wt and *kre6Δ*, the loss rate was strongly reduced in *spc25-1*  *kre6Δ* cells (Fig. 3a), thus supporting the notion that *kre6Δ* suppressed the chromosome segregation defect of *spc25-1*.

We furthermore asked how *kre6Δ* affected the defect of *spc25-1* cells in cell-cycle progression. *spc25-1* cells arrested with a 2n DNA content after 3 h at 30°C, as determined by measuring the DNA content by FACS analysis. In contrast, *spc25-1*  *kre6Δ* cells showed a FACS profile comparable to that of wt cells (Fig. 3b). Interestingly, *kre6Δ* cells also showed a profile similar to that of wt cells, indicating that they do not have a defect in cell-cycle progression, even though they are slightly temperature sensitive (Supplementary Fig. 1a).

Altogether, these results underscored the notion that *kre6Δ* suppresses the chromosome segregation defect of *spc25-1*, and thus, that Kre6 negatively regulates kinetochore function.

### The enzymatic activity of Kre6 is required for its kinetochore function

Kre6 is proposed to be a glycosyl hydrolase or transglycosylase and is required for the production of β-1,6-glucan (Roemer *et al.* 1993). It has been shown to contain the ExDxxE consensus motif (where x designates any amino acid) that is characteristic of such enzymes, and mutation of this sequence to QxNxxQ abrogates Kre6 function (Okada *et al.* 2021). We therefore asked whether the catalytically dead Kre6 mutant (*KRE6^QNQ^*) also suppressed the *spc25-1* temperature sensitivity. Indeed, while plasmid-borne *KRE6* restored poor growth to a *spc25-1*  *kre6*Δ strain at 30°C, *kre6^QNQ^* cells as well as cells carrying an empty vector grew well at 30°C (Fig. 3c). Of note, the protein levels of Kre6 were unaffected by *kre6^QNQ^* (Okada *et al.* 2021), indicating that the absence of complementation was not due to a decrease in Kre6 levels. This showed that the catalytic activity of Kre6 was required for its role in the regulation of kinetochore function.

### A negative role for β-1,6- and β-1,3-glucan metabolism in kinetochore function

**Fig. 4.**
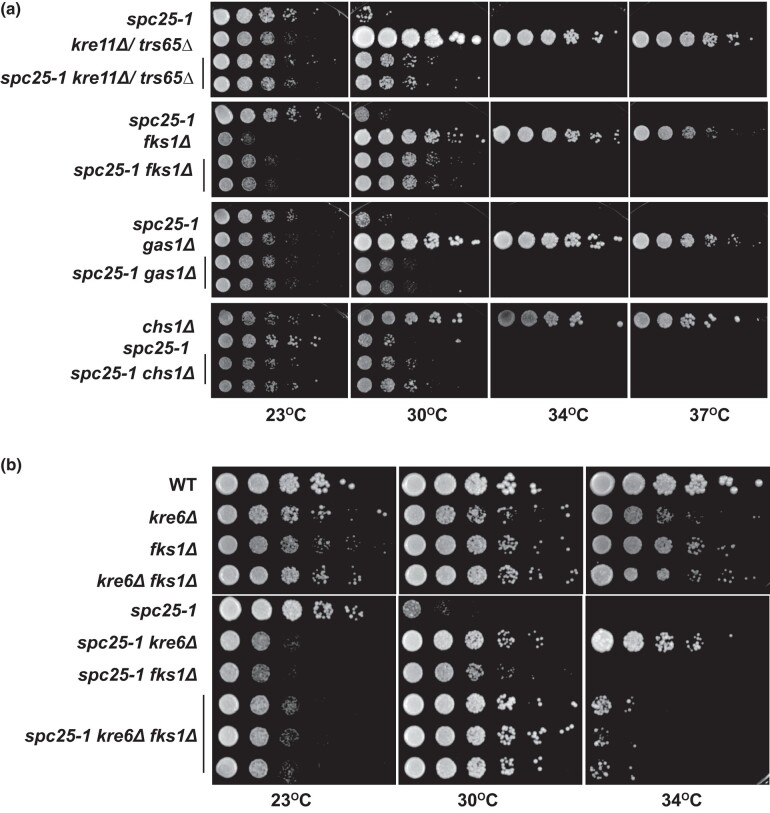
β-1,6- and β-1,3-glucan biosynthesis negatively regulates kinetochore function. a) Deletions of *KRE11/TRS65* and *FKS1* partially suppress the temperature-sensitive growth defect of *spc25-1*, and *gas1Δ* and *chs1Δ* cause a mild suppression. The indicated strains were serially diluted and spotted on YPD medium. Plates were incubated at the indicated temperatures for 3 days. b) The simultaneous reduction of β-1,6- and β-1,3-glucan levels in *kre6Δ fks1Δ* cells causes intermediate suppression of *spc25-1*. Representation as in a).

The involvement of Kre6 in kinetochore function was surprising, given that it so far only has been implicated in synthesis of β-1,6-glucan in the yeast cell wall. To obtain further insights into this, we tested other genes with a role in the synthesis of cell wall components for suppression of *spc25-1*. We first investigated genes with a known role in β-1,6-glucan synthesis (Fig. 1). The deletion of *KRE11*/*TRS65* has previously been shown to cause reduced β-1,6-glucan levels and a smaller polymer size, and *kre11Δ*/*trs65Δ* causes resistance to killer toxin (Brown *et al.* 1993). Kre11/Trs65 is a component of the TRAPPII complex, a so-called tethering complex that mediates the interaction between transport vesicles and their target compartment for the transport of molecules out of the cell (Yip *et al.* 2010). Kre1 is a glycoprotein in the cell wall that functions in the maturation of β-1,6-glucan on the outer surface of the cell (Boone *et al.* 1990). Skn1 is a paralog of Kre6, and high-copy *SKN1* suppresses the growth defect and killer toxin resistance of *kre6Δ*, though *skn1Δ* alone shows no growth defects nor reduced β-1,6-glucan levels (Roemer *et al.* 1993). Of these genes, we found that the deletion of *KRE11*/*TRS65*, but not *SKN1* or *KRE1*, suppressed the temperature sensitivity of *spc25-1* (Fig. 4a, Supplementary Fig. 2a). This indicated that reduced cellular β-1,6-glucan levels, but not matured β-1,6-glucan on the cell surface, were required for the suppression of *spc25-1* kinetochore defects.

We next asked whether a defect in β-1,3-glucan synthesis affects *spc25-1*. Fks1 is a β-1,3-glucan synthase residing in the plasma membrane (Fig. 1), and *fks1Δ* cells have a 75% reduction in β-1,3-glucan levels (Parent *et al.* 1993; Douglas *et al.* 1994; Hu *et al.* 2023). Interestingly, *fks1Δ* caused a pronounced suppression of the *spc25-1* temperature sensitivity (Fig. 4a), though to a slightly lesser degree than *kre6Δ* (Fig. 4b). *fks1Δ* also enhanced the growth defect of *cse4-103* (Supplementary Fig. 2b), as was observed for *kre6Δ*. Gas1 is a plasma membrane protein that cleaves β-1,3-glucosidic linkages within β-1,3-glucan chains and transfers the glycan to another β-1,3-glucan chain (Nuoffer *et al.* 1991). However, *gas1Δ* caused very little, if any, suppression of *spc25-1*, indicating that β-1,3-glucan maturation was at most marginally required for suppression. These findings were consistent with the notion that cellular levels of β-1,3-glucan, rather than extracellular β-1,3-glucan modification, were involved in *spc25-1* suppression and hence kinetochore function.

We were further interested in seeing whether chitin synthesis affected the temperature sensitivity of *spc25-1*. Indeed, the deletion of *CHS1*, which encodes a chitin synthase (Ziman *et al.* 1996), mildly suppressed the *spc25-1* growth defect (Fig. 4a). In contrast, changes in mannosylation levels by deletion of *KRE2*, which encodes a α-1,2-mannosyltransferase (Hausler *et al.* 1992), showed no effect on *spc25-1* (Supplementary Fig. 2a).

Since the absence of Kre6 (β-1,6-glucan synthesis) and Fks1 (β-1,3-glucan synthesis) caused suppression of *spc25-1*, we wondered whether the double deletion *kre6Δ fks1Δ* would cause enhanced suppression, or whether the two deletions would be epistatic to each other. Surprisingly, *spc25-1*  *kre6Δ fks1Δ* cells showed an intermediate phenotype in that they grew better than *spc25-1*  *fks1Δ*, but showed worse growth than *spc25-1*  *kre6Δ* (Fig. 4b). This indicates that the reduction of β-1,6-glucan levels has the strongest effect on *spc25-1*, and that β-1,6-glucan and β-1,3-glucan are partially epistatic to each other with respect to kinetochore function .

In summary, these observations show that cellular β-1,6 and β-1,3-glucan levels are important for suppression of *spc25-1*, whereas chitin levels have a minor effect, and mannose levels do not affect *spc25-1* (Table 2). However, since the maturation of both glucan types seems not to be important (*gas1Δ* has little effect, *kre1Δ* has no effect), this suggests that the presumed role of these glycosylations is not at the cell wall, but more likely a role in an interior compartment of the cell. One possibility is that one or several kinetochore proteins are glycosylated, and that this modification has a negative effect on their function at the kinetochore in the context of a defective NDC80 complex in the *spc25-1* mutant.

**Table 2.** Effect of deletions in cell wall synthesis genes on suppression of *spc25-1*.

Gene	Function	Suppression of *spc25-1^a^*
β-1,6-glucan synthesis		
*KRE6*	Glycosylhydrolase/transglycosidase, β-1,6-glucan biosynthesis	++
*SKN1*	Kre6 homolog, β-1,6-glucan biosynthesis	−
*KRE1*	Cell wall glycoprotein, maturation of β-1,6-glucan	−
*KRE11/TRS65*	Component of TRAPP, deletion causes reduction of β-1,6-glucan levels	+
β-1,3-glucan synthesis		
*FKS1*	β-1,3-glucan synthase	++
*GAS1*	β-1,3-glucanosyltransferase	(+)
Mannosylation		
*KRE2*	α-1,2-mannosyltransferase	−
Chitin synthesis		
*CHS1*	Chitin synthase	(+)

+++, strong suppression, ++ moderate suppression, +, mild suppression, (+) marginal suppression, −, no suppression.

^*a*^Suppression of temperature-sensitive growth defect of *spc25-1*.

### Kre6 interacts in vivo with Cse4^CENP-A^

The above findings indicated that Kre6-mediated β-1,6-glucan levels negatively regulate kinetochore function. A possible scenario is that one or several kinetochore proteins are glycosylated, and that this regulates their function. If so, one prediction is that Kre6 physically interacts with (a) kinetochore protein(s) in the cell. This possibility is supported by earlier work, which found Kre6 by proteomics analysis of IP of a lysine-free version of Cse4^CENP-A^ (all 16 lysines mutated to arginine) (Ranjitkar *et al.* 2010). Intriguingly, in a similar approach for purification of the kinetochore from yeast cells, both Kre6 and Fks1 were retrieved in a precipitation with the MIND component Dsn1 (Akiyoshi *et al.* 2010), thus reinforcing the notion that glucan biosynthesis proteins are associated with the kinetochore.

**Fig. 5.**
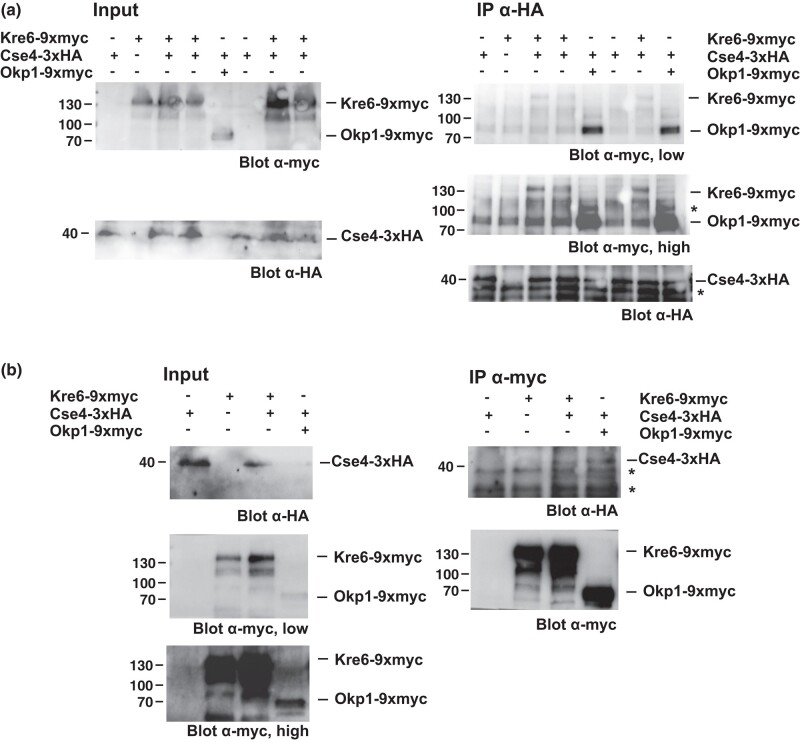
Kre6 interacts in vivo with Cse4. In vivo interaction of Kre6 with Cse4 was determined by co-IP of the proteins from yeast whole-cell extracts. a) HA-tagged Cse4 was precipitated and tested for co-IP of Kre6 and, as a control, of the inner kinetochore protein Okp1. Input (left) and precipitates (IP, right) were subjected to Western blotting with α-HA and α-myc antibody. A low (top right) and high (middle right) exposure of the same α-myc Western blot are shown (see Supplementary Fig. 3a and b for repeat experiments). Unspecific bands are labeled with an asterisk. b) Myc-tagged Kre6 or -Okp1 were precipitated from whole-cell extracts (input) and tested for co-precipitation of 3xHA-tagged Cse4. Low and high exposure blots of the input are shown. Unspecific bands are labeled with an asterisk.

To further test this, we investigated whether Kre6 can co-immunoprecipitate (co-IP) with Cse4^CENP-A^. As a control, co-IP of the inner kinetochore protein Okp1 with Cse4^CENP-A^ was tested (Anedchenko *et al.* 2019). As expected, 9xmyc-tagged Okp1 was detectable in IP of 3xHA-tagged Cse4^CENP-A^, and a low exposure of the Western blot was sufficient for the detection (Fig. 5a, top right). Importantly, a higher exposure of the same blot showed that 9xmyc-tagged Kre6 was precipitated along with Cse4^CENP-A^, and no Kre6 was precipitated in a strain with untagged Cse4^CENP-A^, or when Kre6 was untagged (Fig. 5a, right, middle, see Supplementary Fig. 3a and b for repeat experiments). This indicated that Kre6 interacts with Cse4^CENP-A^ in the cell, but that the interaction may be weaker than that of Cse4^CENP-A^ with Okp1.

We furthermore tested the reverse co-IP, i.e. precipitating Kre6-9xmyc and testing for co-IP of 3xHA-Cse4^CENP-A^. As a control, Okp1 was precipitated, and as expected, Cse4^CENP-A^ was detectable in the precipitate (Fig. 5b, top right). Indeed, in the IP of Kre6, Cse4^CENP-A^ was co-IPed, thus reinforcing the notion that Kre6 interacts with Cse4^CENP-A^ within the cell. This interaction may be direct, or could be mediated by interaction of Kre6 with (an)other kinetochore protein(s).

Since Cse4^CENP-A^ is located at the centromeres, we further asked whether Kre6 also interacts with centromeric sequences. However, in a chromatin immunoprecipitation (ChIP) experiment with Kre6, no enrichment at CEN4 was observed, while Cse4^CENP-A^ was readily ChIPed at CEN4 (Supplementary Fig. 3c). This suggests that Kre6 does not interact with Cse4^CENP-A^ or kinetochore proteins at the centromere itself, but may associate with them in the nucleoplasm or the cytoplasm. Of note, Kre6 is localized to the membrane of several intracellular compartments (Nakamata *et al.* 2007) (Fig. 1).

One hypothesis for the involvement of β-1,6- and β-1,3-glucan biosynthesis in kinetochore biology is that one or several kinetochore proteins carry a glycosyl modification. To investigate this, we attempted to immunoprecipitate Cse4^CENP-A^ with an antibody against β-1,3-glucan (Meikle *et al.* 1991). As a control, Sir2, which has previously been reported to carry this modification (Koch and Pillus 2009), was immunoprecipitated. However, while precipitation of Sir2 was observed, we were unable to IP Cse4^CENP-A^ with the anti-β-1,3-glucan antibody (Supplementary Fig. 3d). This indicates that Cse4^CENP-A^ carries no or only little β-1,3-glucan modification. Alternatively, the anti-β-1,3-glucan antibody, which only recognizes (1→3)-β-oligosaccharide segments in (1→3)-β-glucans, may not be adequate to detect glycosylation on Cse4^CENP-A^.

## Discussion

Posttranslational modifications on proteins are important regulators of protein function. An example in this case is chemical moieties like methyl or acetyl groups on histones that affect the affinity of chromatin-binding proteins and regulate gene expression. Here, we have identified an unexpected role for glycosylation in regulating kinetochore function in *S. cerevisiae*. Specifically, we found that selected genes encoding proteins involved in β-1,6- and β-1,3-glucan synthesis and, to a minor extent, chitin synthesis, played an inhibitory role at the yeast kinetochore in that the absence of the proteins Kre6, Kre11/Trs65, Fks1, and Chs1 suppressed mutations with defects in the outer kinetochore. These findings are unexpected, because these enzymes so far have only been implicated in the synthesis of carbohydrate constituents of the yeast cell wall. Furthermore, we found that Kre6 and the centromeric histone H3 variant Cse4^CENP-A^ interact with each other in yeast cells.

The most direct interpretation of our observations is that Kre6 and Fks1 cooperate to glycosylate Cse4^CENP-A^, and that this negatively regulates its role in kinetochore recruitment and centromere function. The presumed glucan moiety could be a glucose polymer of variable length with β-1,6 and β-1,3 linkages (possibly with a minor contribution of glucosamine). Since Kre6 and Fks1 were negative regulators at the kinetochore, this modification might disturb protein interactions or kinetochore stability. The link to nutritional status through carbohydrate synthesis might provide a connection between nutrient sensing and chromosome segregation. We attempted to test whether Cse4^CENP-A^ is glycosylated by immunoprecipitation with an anti-β-1,3-glucan antibody, but were unsuccessful. Either Cse4^CENP-A^ indeed is not glycosylated, or the variant of modification on Cse4^CENP-A^ is not recognized by the antibody, for instance, if it consists of a short (possibly branched) chain that is not recognized by the antibody. Alternatively, not Cse4^CENP-A^ itself, but a kinetochore protein interacting with Cse4^CENP-A^ is glycosylated by Kre6, a possibility that remains to be tested.

The possible scenario in which Kre6 and Fks1 directly perform glycosylation of Cse4^CENP-A^ is reminiscent of the *O*-linked *N*-acetylglucosamine (O-Glyc-NAc) transferase (OGT) in *Drosophila* (Gambetta *et al.* 2009; Sinclair *et al.* 2009). Mutations in *Drosophila* OGT cause homeotic transformations characteristic of *Polycomb* genes, which arises from a defect in the long-term repression of the *HOX* gene and other developmental regulator genes in the OGT mutant fly embryo. This defect in homeotic gene regulation has been attributed to the O-Glyc-NAcylation of the *Polycomb* repressor protein Polyhomeotic. OGT has also been reported to modify histone H2B in mammalian cells, though this has been critically viewed (Gambetta and Muller 2015). Overall, a plethora of proteins in higher eukaryotes are O-Glyc-NAcylated, including transcription factors, nucleoporins, thus providing a link between nutrient sensing, carbohydrate metabolism, and cell signaling. Therefore, it is conceivable that Cse4^CENP-A^ (or another kinetochore protein) is glycosylated and suggests that this might connect kinetochore function to the nutritional status of the cell. Of note, O-Glyc-NAc does not exist in *S. cerevisiae* (Halim *et al.* 2015). A glycoproteome study investigating O-mannosylation in *S. cerevisiae* identified over 500 proteins carrying this modification, and mannosylated proteins are localized to all subcellular compartments, including the nucleus (Halim *et al.* 2015; Neubert *et al.* 2016).

If Cse4^CENP-A^ indeed is glycosylated, the fact that it is a nuclear protein is not easily reconciled with the subcellular localization of proteins required for cell wall synthesis. Kre6 is found in several secretory compartments, and some Kre6 localizes around the nucleus (Nakamata *et al.* 2007), such that this fraction might be modifying Cse4^CENP-A^. Since we did not find Kre6 associated with the centromere, Kre6 might modify Cse4^CENP-A^ before it is incorporated into the centromeric nucleosome, perhaps in the nucleoplasm, or in the cytoplasm before import into the nucleus. However, Fks1 is a transmembrane protein located in areas of polarized growth (Douglas *et al.* 1994), so it is more difficult to explain how it might modify a nuclear protein.

A functional role for the carbohydrate modification of a nuclear protein is not unprecedented in yeast. There is circumstantial evidence that the silencing protein Sir2 (or an associated factor) is modified with β-1,3-glucan by the β-1,3-glucanosyltransferase Gas1 (see also Supplementary Fig. 3d), and *gas1Δ* causes a telomeric silencing defect, indicating that a carbohydrate modification plays a role in heterochromatin formation at the telomeres (Koch and Pillus 2009). While we found only a minor role for Gas1 at the centromere, a picture emerges in which several cell wall synthesis enzymes have a moonlighting function in chromatin-mediated processes.

While a direct glycosylation of Cse4^CENP-A^ (or another kinetochore protein) is perhaps the most attractive interpretation of our findings, there also are other possibilities. For instance, the absence of the cell wall synthesis proteins could affect cell morphology and mechanical properties of the cell, thus altering intracellular forces that activate cell signaling pathways ultimately impinging on the kinetochore. Alternatively, the levels of cellular metabolites might be altered in the cell wall mutants, and this might then impinge on cell signaling to the kinetochore. Also, since cell wall mutants induce compensatory mechanisms to ensure cellular integrity, indirect effects on other cell wall components or cellular metabolites might affect gene expression, possibly perturbing the levels of centromere proteins. Contrary to this notion, a study of genome-wide changes in gene expression did not identify the effects of cell wall mutants on the expression of kinetochore proteins (Lagorce *et al.* 2003), rendering this scenario less likely. As such, the precise role of carbohydrate synthesis factors in chromosome biology awaits further studies of modifications of kinetochore proteins.

## Supplementary Material

iyad195_Supplementary_Data

## Data Availability

Strains and plasmids are available upon request. Supplementary Tables 1 and 2 list the *S. cerevisiae* strains and plasmids used in this study. The authors affirm that all data necessary for confirming the conclusions of the article are present within the article, figures, and tables. Supplemental material available at GENETICS online.
