# Supplementary material for: A role for β-1,6- and β-1,3-glucans in kinetochore function in Saccharomyces cerevisiae
Source: Genetics. 2023 Nov 10;226(2):iyad195. doi: 10.1093/genetics/iyad195 (PMC11221361; doi:10.1093/genetics/iyad195)
Supplement: iyad195_Supplementary_Data [file iyad195_supplementary_data.pdf]

Supplementary information

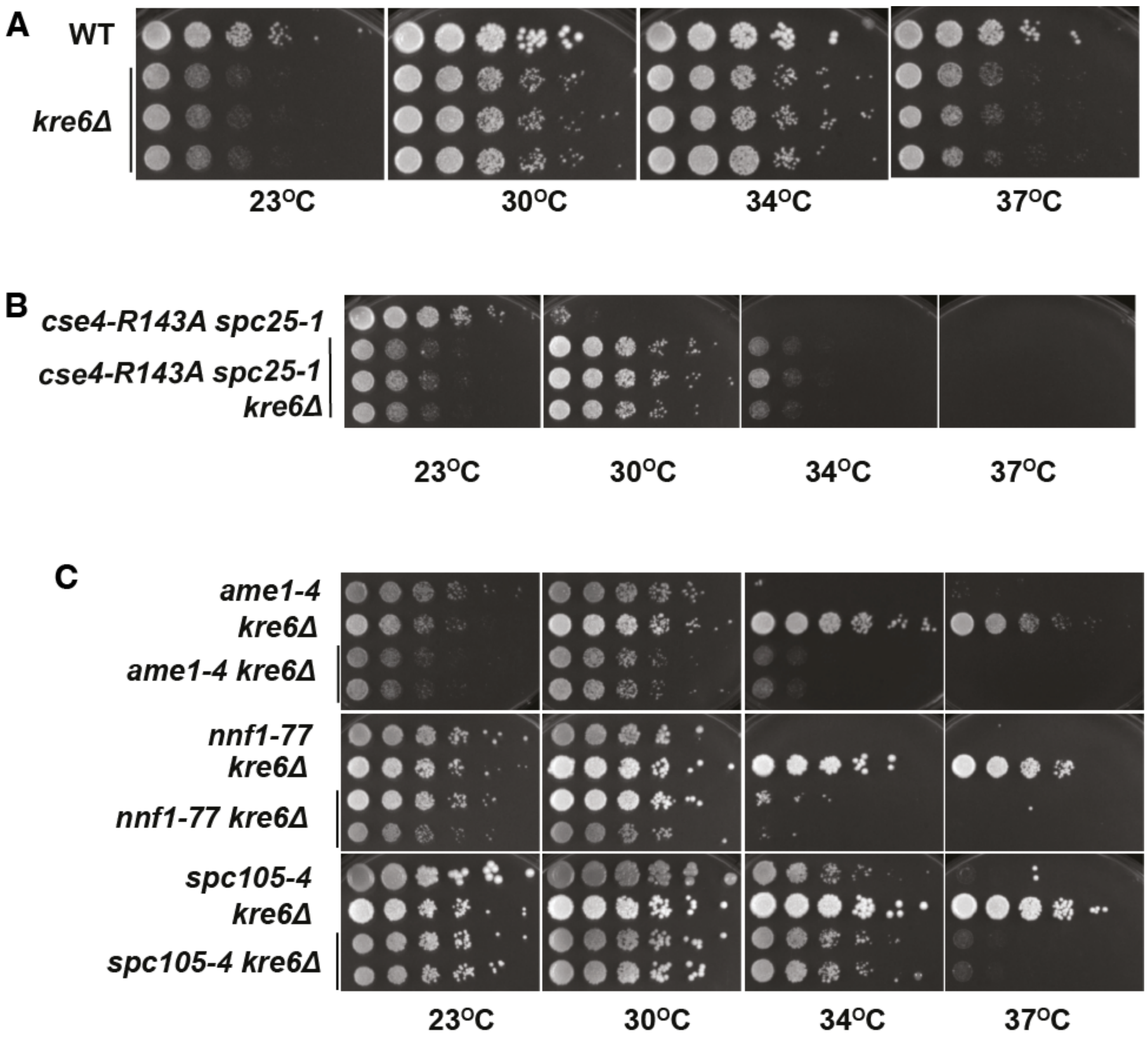

Figure S1: Genetic interactions of *kre6*Δ with mutations in genes encoding kinetochore components. A) *kre6*Δ causes a mild temperature-sensitive growth defect. Representation as in Fig. 2B. B) *kre6*Δ suppressed the temperature sensitivity of *spc25-1 cse4-R143A*. Representation as in Fig. 2B. C) Mutations in *AME1*, *NNF1* and *SPC105* show no genetic interaction with *kre6*Δ. Representation as in Fig. 2B.

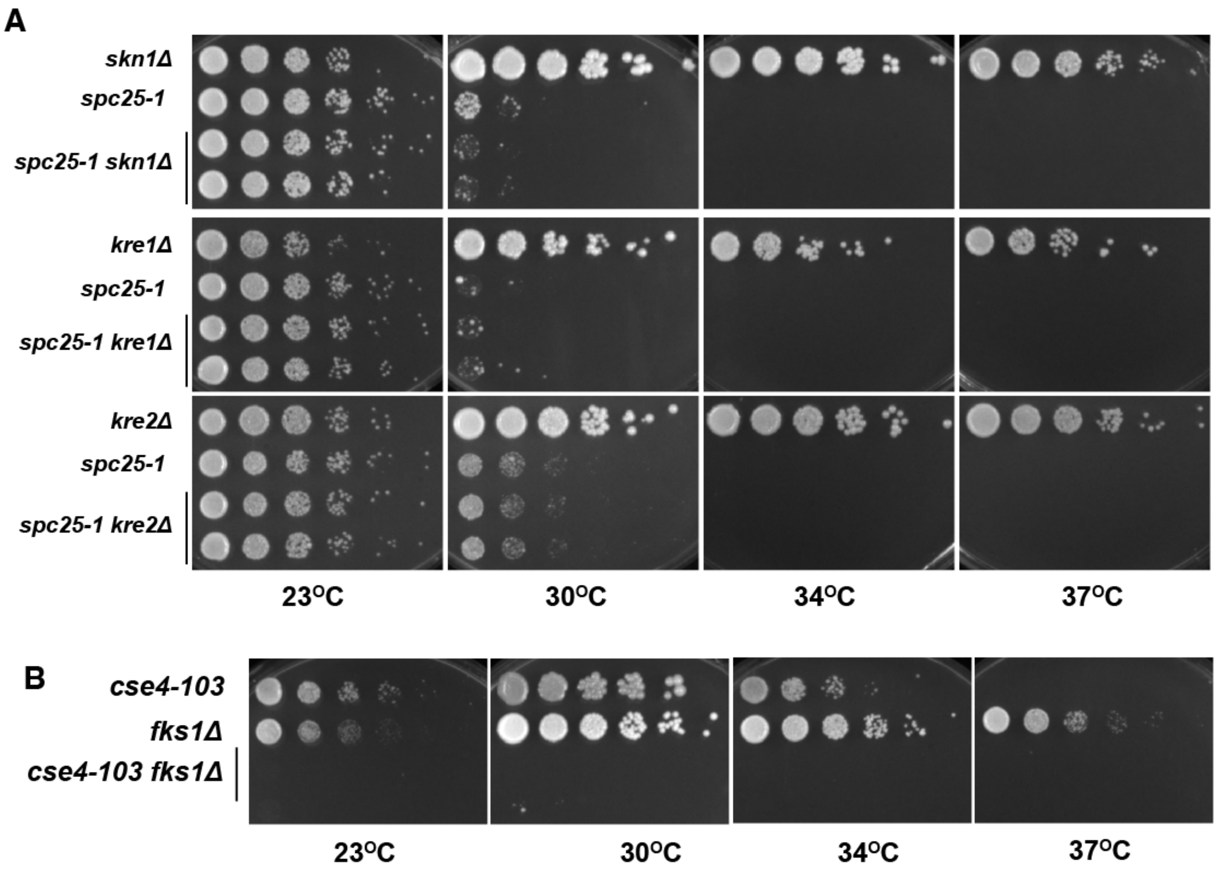

Figure S2: Genetic interactions of *spc25-1* with mutations in genes involved in metabolism of cell wall carbohydrate components. A) *skn1*Δ, *kre1*Δ and *kre2*Δ showed no suppression of *spc25-1*. B) *fks1*Δ enhances the growth defect of *cse4-103*. Representation as in Fig. 2B.

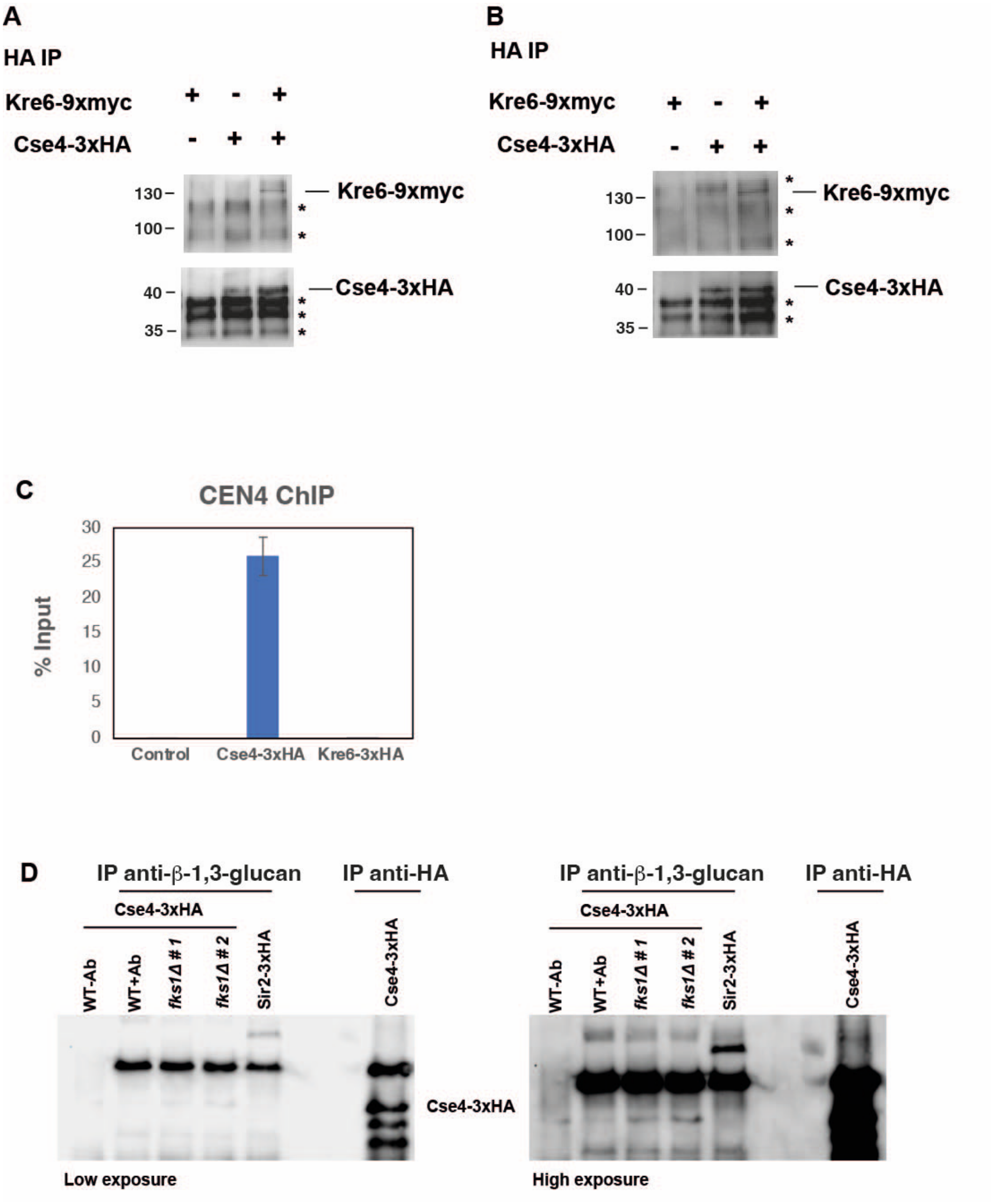

Figure S3: Co-immunoprecipitation of Kre6 and Cse4 from yeast cells. A) and B) Cse4-3xHA was immunoprecipitated from yeast whole-cell extracts, and precipitates were subjected to Western blotting with α-myc (top) and α-HA (bottom) antibodies. Unspecific bands are labelled with an asterisk. C) Kre6 does not ChIP at centromeres. ChIP analysis was performed in wild-type (untagged control), 3xHA-Cse4 and Kre6-6xHA cells. Enrichment at CEN4 relative to input is given (means ± SD, n = 3). D) Cse4 is not precipitated with an anti-β-1,3-glucan antibody. Whole-cell extracts from wt or *fks1*Δ strains with 3xHA-Cse4 and a Sir2-3xHA strain were incubated (+) or not (-) with the anti-β-1,3-glucan antibody. As a control, 3xHA-Cse4 was precipitated with an anti-HA antibody. Cse4 was detected by Western blotting with an anti-HA antibody. Left, low exposure; right, high exposure of the same blot.

Table S1: *S. cerevisiae* strains used in this study

Strain number	Genotype	Source *
AEY 1	*MAT* α *ade2-101 his3-11,15 trp1-1 leu2-3,112 ura3-1 can1-100* (W303)	
AEY 4	*MAT***a** *ADE2 his3-11,15 trp1-1 leu2-3,112 ura3-1 can1-100 lys2Δ* (W303)	
AEY 4758	*MAT* α *cse4Δ::kanMX ade2-101 his3-11,15 trp1-1 leu2-3,112 ura3-1 can1-100 lys2Δ + pTRP1-cse4-103*	
AEY 4922	*MAT***a** *ade2-1 trp1-1 can1-100 leu2-3,112 his3-11,15 ura3 ssd1 ndc80-1*	(NEKRASOV *et al.* 2003)
AEY 4923	*MAT***a** *ade2-1 trp1-1 can1-100 leu2-3,112 his3-11,15 ura3 ssd1 spc24-1*	(NEKRASOV *et al.* 2003)
AEY 4924	*MAT***a** *ade2-1 trp1-1 can1-100 leu2-3,112 his3-11,15 ura3 ssd1 spc25-1*	(NEKRASOV *et al.* 2003)
AEY 4925	*MAT***a** *ade2-1 trp1-1 can1-100 leu2-3,112 his3-11,15 ura3 ssd1 spc105-4*	(NEKRASOV *et al.* 2003)
AEY 5688	*MAT***a** *cse4Δ::kanMX ade2-101 his3-11,15 trp1-1 leu2-3,112 ura3-1 can1-100 lys2Δ* + pRS426-*CSE4-3xHA*	
AEY 6392	*MATa ade2-1 trp1-1 can1-100 leu2-3,112 his3-11,15 ura3 ssd1 dsn1-7*	(NEKRASOV *et al.* 2003)
AEY 6578	*MAT***a** *OKP1-9xMyc::KanMX cbf1Δ::NatMX cse4Δ::HISMX* + pRS426-*CSE4*-3xHA	
AEY 6698	*MAT***a** *okp1-5::TRP1 ade2 LYS2,* W303	
AEY 6700	*MAT***a** *ame1-4::TRP1 ade2 LYS2,* W303	
AEY 6834	*MAT* α *his3-11,15 trp1-1 leu2-3,112 ura3-1 can1-100 ADE2 lys2 cse4-R143A::HISMX spc25-1*	
AEY 7107	*MAT***a** *ADE2 his3-11,15 trp1-1 leu2-3,112 ura3-1 can1-100 lys2Δ skn1Δ::NatMX*	
AEY 7109	*MAT* α *his3-11,15 trp1-1 leu2-3,112 ura3-1 can1-100 ADE lys2 cse4-R143A::HISMX spc25-1 kre6Δ::KanMX*	
AEY 7110	*MAT* α *ADE his3-11,15 trp1-1 leu2-3,112 ura3-1 lys2 kre6Δ::KanMX*	
AEY 7111	*MAT***a** *ADE his3-11,15 trp1-1 leu2-3,112 ura3-1 lys2 kre6Δ::KanMX*	
AEY 7112	*MAT* α *ADE his3-11,15 trp1-1 leu2-3,112 ura3-1 lys2 kre6Δ::KanMX*	
AEY 7113	*MAT***a** *ADE his3-11,15 trp1-1 leu2-3,112 ura3-1 lys2 cse4-R143A::HISMX spc25-1 kre6Δ::KanMX*	
AEY 7114	*MAT* α *ADE his3-11,15 trp1-1 leu2-3,112 ura3-1 lys2 cse4-R143A::HISMX spc25-1 kre6Δ::KanMX*	
AEY 7116	*MAT***a** *ADE his3-11,15 trp1-1 leu2-3,112 ura3-1 lys2 spc25-1 kre6Δ::KanMX*	
AEY 7117	*MAT* α *ADE his3-11,15 trp1-1 leu2-3,112 ura3-1 lys2 spc25-1*	
AEY 7118	*MAT* α *ade2 his3-11,15 trp1-1 leu2-3,112 ura3-1 lys2 spc25-1 kre6Δ::KanMX*	
AEY 7119	*MAT***a** *ADE2 his3-11,15 trp1-1 leu2-3,112 ura3-1 can1-100 lys2Δ kre2Δ::NatMX*	
AEY 7125	*MAT* α *ade2 his3-11,15 trp1-1 leu2-3,112 ura3-1 lys2 spc105-4 kre6Δ::KanMX*	
AEY 7126	*MAT***a** *ade2 his3-11,15 trp1-1 leu2-3,112 ura3-1 lys2 spc105-4 kre6Δ::KanMX*	
AEY 7127	*MAT***a** *ade2 his3-11,15 trp1-1 leu2-3,112 ura3-1 LYS2 spc24-1 kre6Δ::KanMX*	
AEY 7128	*MAT* α *ADE his3-11,15 trp1-1 leu2-3,112 ura3-1 LYS2 spc24-1 kre6Δ::KanMX*	
AEY 7129	*MAT* α *ade2 his3-11,15 trp1-1 leu2-3,112 ura3-1 LYS2*	

	ndc80-1 kre6Δ::KanMX	
AEY 7130	MATa ade2 his3-11,15 trp1-1 leu2-3,112 ura3-1 LYS2 ndc80-1 kre6Δ::KanMX	
AEY 7131	MATa ADE his3-11,15 trp1-1 leu2-3,112 ura3-1 lys2 dsn1-7 kre6Δ::KanMX	
AEY 7132	MAT α ADE his3-11,15 trp1-1 leu2-3,112 ura3-1 lys2 dsn1-7 kre6Δ::KanMX	
AEY 7133	MATa ADE his3-11,15 trp1-1 leu2-3,112 ura3-1 lys2 spc25-1 skn1Δ::NatMX	
AEY 7134	MAT α ADE his3-11,15 trp1-1 leu2-3,112 ura3-1 lys2 spc25-1 skn1Δ::NatMX	
AEY 7135	MAT α ADE his3-11,15 trp1-1 leu2-3,112 ura3-1 lys2 spc25-1 kre2Δ::NatMX	
AEY 7136	MATa ADE his3-11,15 trp1-1 leu2-3,112 ura3-1 lys2 spc25-1 kre2Δ::NatMX	
AEY 7137	MAT α ADE his3-11,15 trp1-1 leu2-3,112 ura3-1 lys2 okp1-5::TRP1 kre6Δ::KanMX	
AEY 7138	MATa ade2 his3-11,15 trp1-1 leu2-3,112 ura3-1 LYS2 okp1-5::TRP1 kre6Δ::KanMX	
AEY 7139	MAT α ADE his3-11,15 trp1-1 leu2-3,112 ura3-1 lys2 ame1-4::TRP1 kre6Δ::KanMX	
AEY 7140	MATa ADE his3-11,15 trp1-1 leu2-3,112 ura3-1 lys2 ame1-4::TRP1 kre6Δ::KanMX	
AEY 7141	MATa ADE2 his3-11,15 trp1-1 leu2-3,112 ura3-1 can1-100 lys2Δ fks1Δ::NatMX	
AEY 7172	MATa ADE2 his3-11,15 trp1-1 leu2-3,112 ura3-1 can1-100 lys2Δ kre11Δ::KanMX	
AEY 7177	MAT α ade2-101 his3-11,15 trp1-1 leu2-3,112 ura3-1 can1-100 KRE6::9xmyc KanMX	
AEY 7179	MAT α ade2-101 his3-11,15 trp1-1 leu2-3,112 ura3-1 can1-100 gas1Δ::HygR	
AEY 7183	MAT α ade2-101 his3-11,15 trp1-1 leu2-3,112 ura3-1 can1-100 KRE6::6xHA HygR	
AEY 7185	MAT α ADE2 his3-11,15 trp1-1 leu2-3,112 ura3-1 can1-100 lys2Δ spc25-1 fks1Δ::NatMX	
AEY 7186	MATa ADE2 his3-11,15 trp1-1 leu2-3,112 ura3-1 can1-100 lys2Δ spc25-1 fks1Δ::NatMX	
AEY 7188	MATa ADE2 his3-11,15 trp1-1 leu2-3,112 ura3-1 can1-100 lys2Δ fks1Δ::NatMX Kre6Δ::KanMX	
AEY 7189	MATa ADE2 his3-11,15 trp1-1 leu2-3,112 ura3-1 can1-100 lys2Δ spc25-1 fks1Δ::NatMX kre6Δ::KanMX	
AEY 7190	MAT α ADE2 his3-11,15 trp1-1 leu2-3,112 ura3-1 can1-100 lys2Δ spc25-1 fks1Δ::NatMX kre6Δ::KanMX	
AEY 7191	MAT α ADE2 his3-11,15 trp1-1 leu2-3,112 ura3-1 can1-100 lys2Δ spc25-1 fks1Δ::NatMX kre6Δ::KanMX	
AEY 7197	MATa ADE2 his3-11,15 trp1-1 leu2-3,112 ura3-1 lys2 spc25-1 kre11Δ::KanMX	
AEY 7198	MATa ADE2 his3-11,15 trp1-1 leu2-3,112 ura3-1 lys2 spc25-1 kre11Δ::KanMX	
AEY 7207	MAT α cse4Δ::kanMX ade2-101 his3-11,15 trp1-1 leu2-3,112 ura3-1 can1-100 KRE6-9xmyc::KanMX pRS426-CSE4-3xHA	
AEY 7212	MAT α cse4Δ::kanMX ade2-101 his3-11,15 trp1-1 leu2-3,112 ura3-1 can1-100 lys2 kre6Δ::KanMX + pRS426-CSE4-3xHA	
AEY 7239	MAT α ade2-101 his3-11,15 trp1-1 leu2-3,112 ura3-1 can1-100 chs1Δ::NatMX	
AEY 7241	MATa ade2-101 trp1-1 can1-100 leu2-3,112 his3-11,15 ura3 spc25-1 gas1Δ::HygrB	
AEY 7242	MATa ade2-101 trp1-1 can1-100 leu2-3,112 his3-11,15 ura3 spc25-1 gas1Δ::HygrB	

AEY 7244	*MAT***a** *ade2-101 trp1-1 can1-100 leu2-3,112 his3-11,15 ura3 spc25-1 chs1Δ::NatMX*	
AEY 7245	*MAT***a** *ade2-101 trp1-1 can1-100 leu2-3,112 his3-11,15 ura3 spc25-1 chs1Δ::NatMX*	
AEY 7254	*MAT α cse4Δ::kanMX ade2-101 his3-11,15 trp1-1 leu2-3,112 ura3-1 can1-100 lys2 kre6Δ::KanMX+ pRS313-cse4-103*	
AEY 7255	*MAT α cse4Δ::kanMX ade2-101 his3-11,15 trp1-1 leu2-3,112 ura3-1 can1-100 lys2 kre6Δ::KanMX # 2 + pAE1510 pRS313-cse4-103*	
AEY 7257	*MAT***a** *cse4Δ::kanMX ade2-101 his3-11,15 trp1-1 leu2-3,112 ura3-1 can1-100 lys2Δ fks1Δ::NatMX + pAE977 pRS426-Cse4-3xHA*	
AEY 7258	*MAT***a** *cse4Δ::kanMX ade2-101 his3-11,15 trp1-1 leu2-3,112 ura3-1 can1-100 lys2Δ fks1Δ::NatMX + pAE977 pRS426-Cse4-3xHA*	
AEY 7259	*MAT***a** *cse4Δ::kanMX ADE2 his3-11,15 trp1-1 leu2-3,112 ura3-1 can1-100 lys2Δ fks1Δ::NatMX + pAE977 pRS426-Cse4-3xHA*	
AEY 7260	*MAT***a** *ADE2 his3-11,15 trp1-1 leu2-3,112 ura3-1 can1-100 lys2Δ kre1Δ::KanMX*	
AEY 7262	*MAT***a** *ADE his3-11,15 trp1-1 leu2-3,112 ura3-1 LYS2 nnf1-77 kre6Δ::KanMX*	
AEY 7263	*MAT***a** *ade2 his3-11,15 trp1-1 leu2-3,112 ura3-1 LYS2 nnf1-77 kre6Δ::KanMX*	
AEY 7264	*MAT***a** *ade2 his3-11,15 trp1-1 leu2-3,112 ura3-1 lys2 nsl1-6 kre6Δ::KanMX*	
AEY 7265	*MAT α ade2 LYS2 his3-11,15 trp1-1 leu2-3,112 ura3-1 lys2 mtw1-11 kre6Δ::KanMX*	
AEY 7270	*MAT***a** *cse4Δ::kanMX ADE2 his3-11,15 trp1-1 leu2-3,112 ura3-1 can1-100 lys2Δ fks1Δ::NatMX + pRS313-cse4-103*	
AEY 7271	*MAT***a** *cse4Δ::kanMX ADE2 his3-11,15 trp1-1 leu2-3,112 ura3-1 can1-100 lys2Δ fks1Δ::NatMX + pRS313-cse4-103*	

* Unless indicated otherwise, strains were from the laboratory collection or were constructed in the course of this study. All strains are derivatives of W303.

Table S2: Plasmids used in this study

Plasmid	Description	Source *
pAE261	pRS315	(SIKORSKI AND HIETER 1989)
pAE977	pRS426-*CSE4*-3xHA	
pAE1510	pRS313-*cse4-103*	
pAE3650	pRS315-ENVY-KRE6	(OKADA *et al.* 2021)
pAE3651	pRS315-ENVY-KRE6 QNQ	(OKADA *et al.* 2021)

* Plasmids were from the laboratory collection or constructed in the course of this study, unless indicated otherwise.

REFERENCES

Nekrasov, V. S., M. A. Smith, S. Peak-Chew and J. V. Kilmartin, 2003 Interactions between centromere complexes in Saccharomyces cerevisiae. Mol Biol Cell 14**:** 4931-4946.

Okada, H., B. MacTaggart, Y. Ohya and E. Bi, 2021 The kinetic landscape and interplay of protein networks in cytokinesis. iScience 24**:** 101917.

Sikorski, R. S., and P. Hieter, 1989 A system of shuttle vectors and yeast host strains designed for efficient manipulation of DNA in Saccharomyces cerevisiae. Genetics 122**:** 19-27.
